# Accuracy of final canine retraction with and without a palatal power arm using in-house clear aligners: a randomized clinical trial

**DOI:** 10.1186/s40510-026-00630-5

**Published:** 2026-06-23

**Authors:** Sakda Wonghinkong, Natnicha Vongtiang, Sawitt Eurutairat, Somchai Manopatanakul, Peerapong Santiwong, Nita Viwattanatipa

**Affiliations:** Department of Orthodontics, Faculty of Dentistry, Mahidol University, Bangkok, Thailand

**Keywords:** Equivalence test, Canine retraction, Clear aligner, Tooth movement, Premolar extraction

## Abstract

**Objectives:**

To evaluate the accuracy of maxillary canine and anchorage tooth movement in first premolar extraction cases at final canine retraction, using In-house clear aligners (IHCA), by comparing the palatal power arm (PA) to control (C).

**Methods and Design:**

A single-center randomized controlled trial with a split-mouth design was used. *Setting*: University. *Participants and interventions*: Eighteen adults requiring extraction of maxillary first premolars were recruited and received multi-stage IHCA treatment. *Main outcome measure:* Outcomes include 3 linear (mesio-distal, bucco-lingual, extrusion-intrusion) and 3 angular (tipping, rotation, torque) measurements. The primary outcome was canine distalization. *Sequence generation:* Patients were assigned to the PA and control sides using sequence-based randomization (Research-Randomizer website). Allocation concealment and blinding were not feasible. Pretreatment and canine final aligner virtual and actual digital models were superimposed using the 3D GOM-Inspect-Suite software to assess 6 types of tooth movement. Equivalence testing with Holm–Bonferroni correction was used to compare mean differences and 90% confidence intervals (CI) between PA and control sides for maxillary canines and anchorage teeth.

**Results:**

For canine linear movements, the mean differences ranged from −0.19 to 0.09 mm, with corresponding 90% confidence intervals entirely within the predefined equivalence margins of ± 0.5 mm, indicating practical equivalence. In contrast, canine rotation was not equivalent, with a mean difference of 5.22° and a 90% CI of (1.48, 8.95), which exceeded the predefined equivalence bounds of ± 1.5°. Similarly, canine distal crown tipping failed to demonstrate equivalence, with a mean difference of 1.67° and a 90% CI of (− 0.44, 3.78). For anchorage movements, most linear outcomes met the equivalence criteria, except for premolar mesialization and molar buccalization, whereas angular outcomes generally did not, as their confidence intervals were not fully contained within the equivalence region. These findings suggest loss of anchorage on both sides, characterized by mesial crown tipping and relative intrusion, indicating that the PA may not significantly preserve anchorage.

**Conclusions:**

Linear movements were equivalent between the PA and control, whereas canine angular movements did not demonstrate equivalence. Potential benefit of PA in angulation and rotation control should be interpreted with caution. These findings may have limited generalizability, especially to commercial aligners.

*Trial Registration* Current Controlled Trials ISRCTN 14020146 of the International Standard Randomized Controlled Trial.

## Introduction

### Background

Modern clear aligner treatment is a practical option for complex malocclusions [1]. Early studies focused on simple, non-extraction cases [2, 3]. Recently, clinicians have evaluated the predictability of aligners in complex cases. New research highlights factors affecting accuracy, such as case complexity, misalignment severity, treatment planning, digital scans, material properties, interproximal reduction, attachments, clinician experience, and patient cooperation etc. [4–10].

Treating extraction cases with clear aligners is challenging because root paralleling is inconsistent after space closure [11–13]. Recent research primarily focuses on Invisalign, with outcomes categorized as predictability (difference, accuracy) [12–16] and evaluations of treatment results in randomized controlled trials (RCTs) using ABO-OGS [17] or the PAR index [10].

Many clinicians and researchers have noted the occurrence of the “roller coaster effect” or “bowing effect” in extraction cases [18, 19]. Previous clinical studies have highlighted significant side effects during space closure, including lingual tipping and extrusion of upper incisors, distal tipping and extrusion of canines, as well as mesial tipping and intrusion of premolars and molars, indicating a loss of anchorage [14–16, 20]. These findings indicate that the actual tooth movements do not align with the predictions in the virtual plan, necessitating supplementary tools such as attachments, inter-arch elastics, IPR, mini-screws, and modifications to aligner designs to enhance the predictability of treatment outcomes [6].

Uncontrolled tipping during canine retraction occurs because the aligner force is typically applied below the tooth’s center of resistance, limiting root control. Introducing a power arm can modify the point of force application and increase the moment-to-force ratio, thereby enabling more controlled tooth movement. Gaffuri [17] and Womack [21] recommended using power arms on canines to improve control of root angulation during retraction. Applying this biomechanical principle to clear aligner therapy may therefore enhance control of canine tipping during extraction space closure.

In-house clear aligners (IHCA) have gained attention among orthodontists. They enable clinicians to control the fabrication and treatment processes, customizing protocols for better outcomes. Studies on IHCA’s effectiveness, especially in extraction cases, are limited [22]. Few examine premolar extraction. Jaber et al. [10] compared the PAR index between IHCA and fixed appliances in four premolar extractions but did not assess tooth movement accuracy. The clinical accuracy of IHCA in extractions remains underexplored. Our research question focuses on the accuracy of maxillary canine movement.

### Specific objectives or hypotheses

Our goal was to evaluate the effectiveness of tooth movement using IHCA in adults requiring extraction of the maxillary first premolar, with a focus on final maxillary canine retraction and anchorage. The primary goal of our study was to evaluate the accuracy of 6 types of canine tooth movement between the palatal power arm (PA) side and the non-power arm (C) control side, using the equivalence test. The null hypothesis was that the mean paired difference between the palatal power arm and control sides exceeded the prespecified equivalence margin, indicating clinical non-equivalence.

## Methods

### Trial design

This investigation was conducted as a prospective, single-center randomized controlled trial using a split-mouth design with a 1:1 allocation ratio. No changes after trial commencement.

### Participants, eligibility criteria, and study setting

Ethical approval was obtained from the Institutional Review Board (IRB) of the Faculty of Dentistry and Faculty of Pharmacy (DTPY-IRB), Mahidol University, and the study was registered with the International Standard Randomized Controlled Trial registry (ISRCTN 14020146). The trial was designed and reported following the CONSORT 2010 guidelines. Adult orthodontic patients (age > 18 years old) undergoing IHCA treatment at the Orthodontic Clinic, Faculty of Dentistry, Mahidol University, were enrolled in this clinical study from 2020 to 2023. The details of the inclusion and exclusion criteria are listed in Table 1.

**Table 1:** Inclusion and exclusion criteria [23, 27]

Inclusion	Exclusion
1) Aged 18 years or older.	1) Moderate to severe rotation of maxillary canine position (Deviation from normal alignment > 20°).
2) Angle Class I or II, with maxillary anterior teeth proclination /protrusion.	2) Asymmetrical position of maxillary right and left canine (Anteroposterior asymmetry > 2 mm).
3) Maxillary arch crowding ranges from none to mild (< 3 mm).	3) Missing maxillary permanent teeth except 3rd molars.
4) Requiring comprehensive treatment for all permanent teeth, including bilateral symmetrical extraction of the maxillary first premolars.	4) Active periodontal disease. 5) Non-compliance.
5) Treatment plan requiring maximum anchorage of the maxillary arch (Anchorage loss < 25% of extraction space site).	6) Pathologies. i. Presence of systemic illnesses and/or bone related diseases. ii. On medication which could impact on study outcomes such as receiving anti-resorptive or anti-angiogenic drugs. iii. History of receiving head and neck radiotherapy. iii. Hypercementosis and/or any dental anomalies.
	7) Pregnancy. 8) Patients planning to relocate or move during the treatment period.

The research design used in this study follows the methodology described in our previously published articles by Vongtiang et al. [23].

### Interventions

#### Pretreatment orthodontic protocol and intraoral scanning

**Fig. 1:**
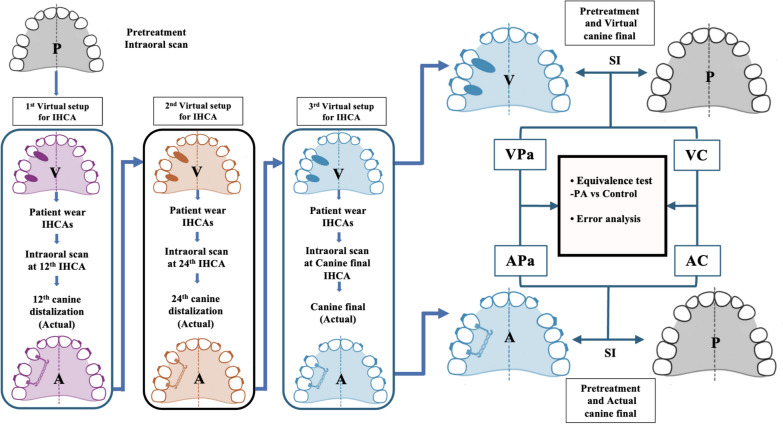
Research design Abbreviation; In-house clear aligner (IHCA), virtual (V), actual (A), pretreatment (P), superimposition (SI), virtual-power arm (VPa), virtual-control (VC), actual-power arm (APa) and actual-control (AC)

Pretreatment data were collected. The research design of our study is shown in Fig. 1. Sequential intraoral scanning was performed at the pretreatment stage, during treatment, and after achieving final canine retraction using an iTero scanner (Align Technology, Inc., San Jose, CA, USA). The research advisors (NV and SM) made the diagnosis and treatment plan for each case.

#### In-house laboratory workflow

The protocol for our IHCA fabrication process to achieve final maxillary canine retraction was divided into multiple stages (Fig. 1; Table 2). In each stage, the canines were moved by 3 mm per stage (0.5 mm per model), except for the final stage, which may vary depending on the remaining space.

**Table 2:** Compensation tooth setup protocol of each tooth in maxillary arch for canine final model [23, 25, 27]

Tooth	Linear movement	Angulation	Extrusion /Intrusion
*Stage 1* which includes the 1st – 12th aligners			
Canine	Distalized 3 mm	Distal root tip 8°-10°	0 mm
2nd premolar	–	Mesial root tip 5–7°	Ext 0.7 mm
1st molar	–	Mesial root tip 5°	Ext 0.5 mm
*Stage 2* which includes the 13th – 24th aligners			
Canine	Distalized 3 mm	Distal root tip 8°-10°	0 mm
2nd premolar	–	Mesial root tip 5–7°	Ext 0.7 mm
1st molar	–	Mesial root tip 5°	Ext 0.5 mm
*Stage 3* which includes the 25th – Canine final aligner*			
Canine	Depend on residual space	Distal root tip 8°-10°	0 mm
2nd premolar	–	Mesial root tip 5–7°	Ext 0.7 mm
1st molar	–	Mesial root tip 5°	Ext 0.5 mm

^*^The canine final aligners varied for each patient depending on the residual space

#### Digital model preparation and virtual tooth movement

For model setup preparation, STL-scanned files from pretreatment and the start of each stage were imported into Ortho-Analyzer software (3Shape, Copenhagen, Denmark). The general protocol for tooth movement in this study was adapted from Lombardo et al. [25]. The compensation tooth setup protocol is shown in Table 2, and the lab and clinical protocols are in Table 3.

**Table 3:** Laboratory and clinical protocol of In-house clear aligner treatment [23, 25, 27]

*Laboratory Protocol*	
Linear movement in combined A-P/ Vertical/ Transverse direction	0.5 mm per model or 0.25 mm per aligner [25]
Mesiodistal tip and rotation	Not exceeding 2° per model or 1° per aligner [25]
Torque	Not exceeding 1° per model or 0.5° per aligner [25]
Printing Protocols	Each model was used to construct 2 aligners with different in thickness. 0.5 mm and 0.75 mm Stage 1: 6 printed models and 1 model template for attachment placement Stage 2: 6 printed models and 1 model template for attachment placement Stage 3: A number of printed model and 1 model template for attachment placement
Gingival trimline	2-mm gingival extension
*Clinical Protocol*	
All stages	**Auxiliaries** - Palatal power arm - 12 mm in length on experiment canine - 8 mm in length on experiment first molar - Super-elastic power chains connected between the palatal power arm (change every 6–8 weeks) - Buttons UL3/ UR3/ LL6/ LR6. Button LL5 and LR5 starting from stage 2. - Reinforced anchorage using Class II elastics 80-100 g of force (3/16″ 2–3.5 ounces) **Instruction** - Aligners must be worn at least 22 h a day. - Aligners change was every 1 week/ piece - Instruction of using chewy twice a day **Follow up**: every 6–8 weeks **Off track management** - Back track - Elastic traction - Rescan

Both the PA and control sides received the same aligner protocol, including identical staging design, attachment design, tooth movement setup, and aligner sequence. On the PA side, a palatal power arm was attached to the maxillary canine and connected to the maxillary first molar with a superelastic power chain to deliver distalizing force. On the control side, canine distalization was achieved using the aligner system alone, without a palatal power arm or superchain. Thus, the only difference between the two sides was the presence of the palatal power arm and the additional force delivered by the superchain.

#### Attachment/power arm

**Fig. 2:**
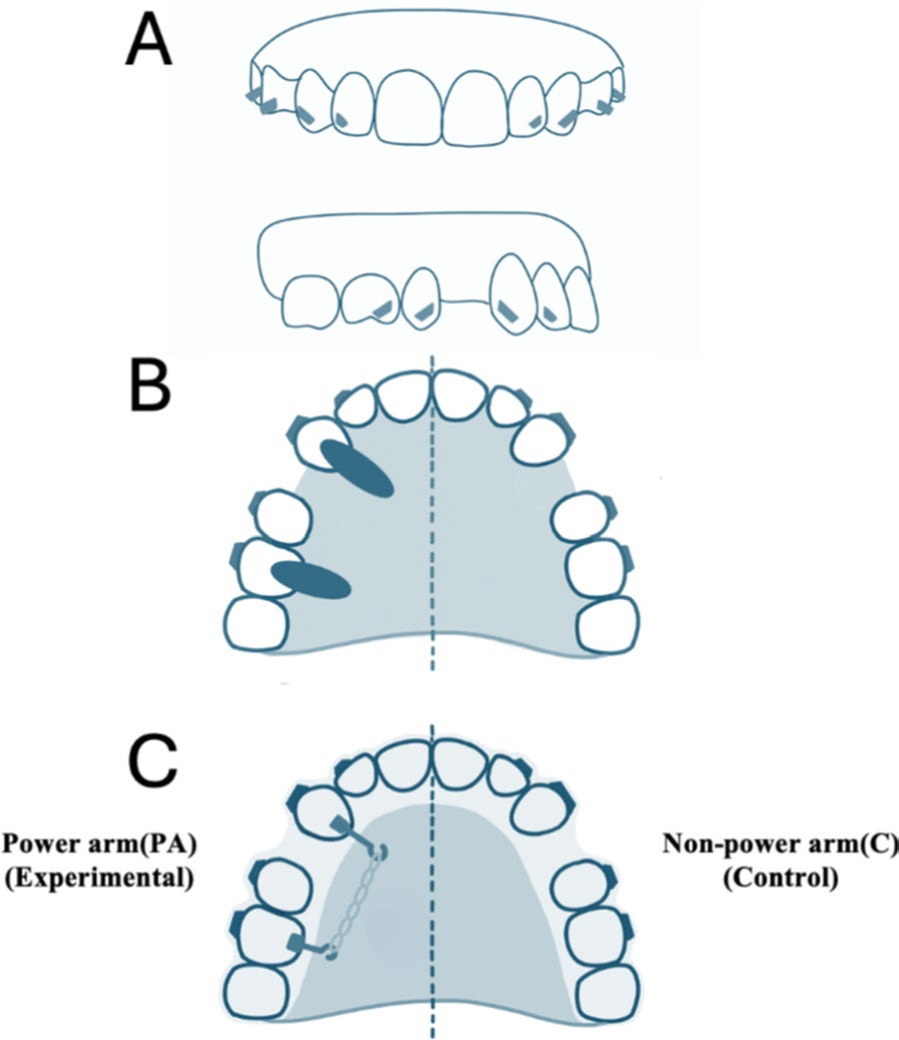
Attachment design and power arm placement **A** Location of triangular prism-shaped attachments; **B** Long-oval shape of block-out area for power arm placement on canine and first molar at experimental side; **C** In-house clear aligner (IHCA), the power arm (PA) and control (C)

Three-sided prism attachments were bonded bilaterally to the maxillary canines, second premolars, first molars, and lateral incisors. IHCA with the power arm (PA) on the maxillary canine and the ipsilateral first molar on the experimental side is shown in Fig. 2.

### Model printing

Once the setup was complete, it was divided into six models and one template for the power arm's attachment and housing. The digital models were then exported to Meshmixer (Autodesk Inc., San Francisco, CA, USA) to prepare them as hollow base models with a 3-mm shell thickness. The printing protocol followed that of Vontiang et al. [23], Tongkitcharoen et al. [26], and Eurutairat et al. [27].

### Thermoforming of clear aligners

IHCA were thermoformed using polyethylene terephthalate glycol (PET-G) thermoplastic sheets of 0.5 and 0.75 mm (3A-MEDES, Gyeonggi-do, Republic of Korea) in a pressure molding device (Biostar®; Scheu Dental, Iserlohn, Germany), with each aligner set consisting of two sequential aligners 0.5 mm to facilitate initial tooth movement and aligner seating, followed by 0.75 mm to increase appliance stiffness and enhance biomechanical control [28].

### Clinical procedure

#### Power arm and attachment bonding (Fig. 2)

Prefabricated power arms (W&H Tech Co., Ltd., Bangkok, Thailand) were bonded to the cervical one-third of the palatal surfaces of the maxillary canines (12 mm) and the corresponding first molars (8 mm) on the experimental side, together with the placement of the attachments.

Following our standardized extraction protocol, both maxillary first premolars were removed on the same day, and the IHCA appliances were delivered within one week thereafter. A superelastic power chain delivering 80–100 g of force (TOMY Inc., Tokyo, Japan) was connected between the power arms on the experimental side and replaced every 6–8 weeks. The force delivered by the power chain was measured using an orthodontic force gauge (DENTAURUM GmbH & Co. KG, Ispringen, Germany) prior to clinical application.

The aligner-wearing and follow-up protocol is presented in Table 3.

### Outcomes

- Data collection

Intraoral scanning was performed after the maxillary canine final aligner was completed, and this was defined as the actual data. The STL files exported from the maxillary canine final setup were considered the virtual data. Baseline data and patient characteristics were collected.

b. Deviation Analysis

Accuracy assessment of the clear aligners was conducted through deviation analysis using the GOM Inspect Suite software (Carl Zeiss GOM Metrology, Braunschweig, Germany). The primary and secondary outcome variables, including their definitions and abbreviations, are summarized in Table 4.

**Table 4:** Definition and abbreviation terms of parameters and outcome measurement in maxillary model superimposition [23, 27]

*Parameters*		
Tooth		Canine Second premolar First molar
Axis referral		X = Transverse Y = Occluso-gingival Z = Antero-posterior
Surface points on model	Palate	Palate references area of 3 points (R1, R2, and R3) R1: is the point on the third rugae of the left side, located at the intersection of the horizontal and vertical slopes of the palate. R2: is the point on the medial end of the third rugae on the left side, which is closest to the midpalatal raphe. R3: is the point on the third rugae of the right side, located at the intersection of the horizontal and vertical slopes of the palate
	Canine	M = mesial point angle I = cusp tip D = distal point angle G = center of gingival surface at buccal
	Second Premolar	B = buccal cusp MB = mesio-buccal point angle DB = disto-buccal point angle G = center of gingival surface at buccal
	First molar	B = tip end of buccal groove MB = mesio-buccal cusp tip DB = disto-buccal cusp tip G = center of gingival surface at buccal
Angulation on model	Tip	Angle between two B/I – G lines in YZ Axis
	Torque	Angle between two B/I – G lines in XY Axis
	Rotation	Angle between two M—D lines in XZ Axis
Primary Outcomes		Canine distalization
Secondary Outcomes		Mesio-Distal displacement Bucco-Lingual displacement Extrusion-Intrusion displacement Tipping Torque Rotation

#### Model superimposition (Fig. 3)

**Fig. 3:**
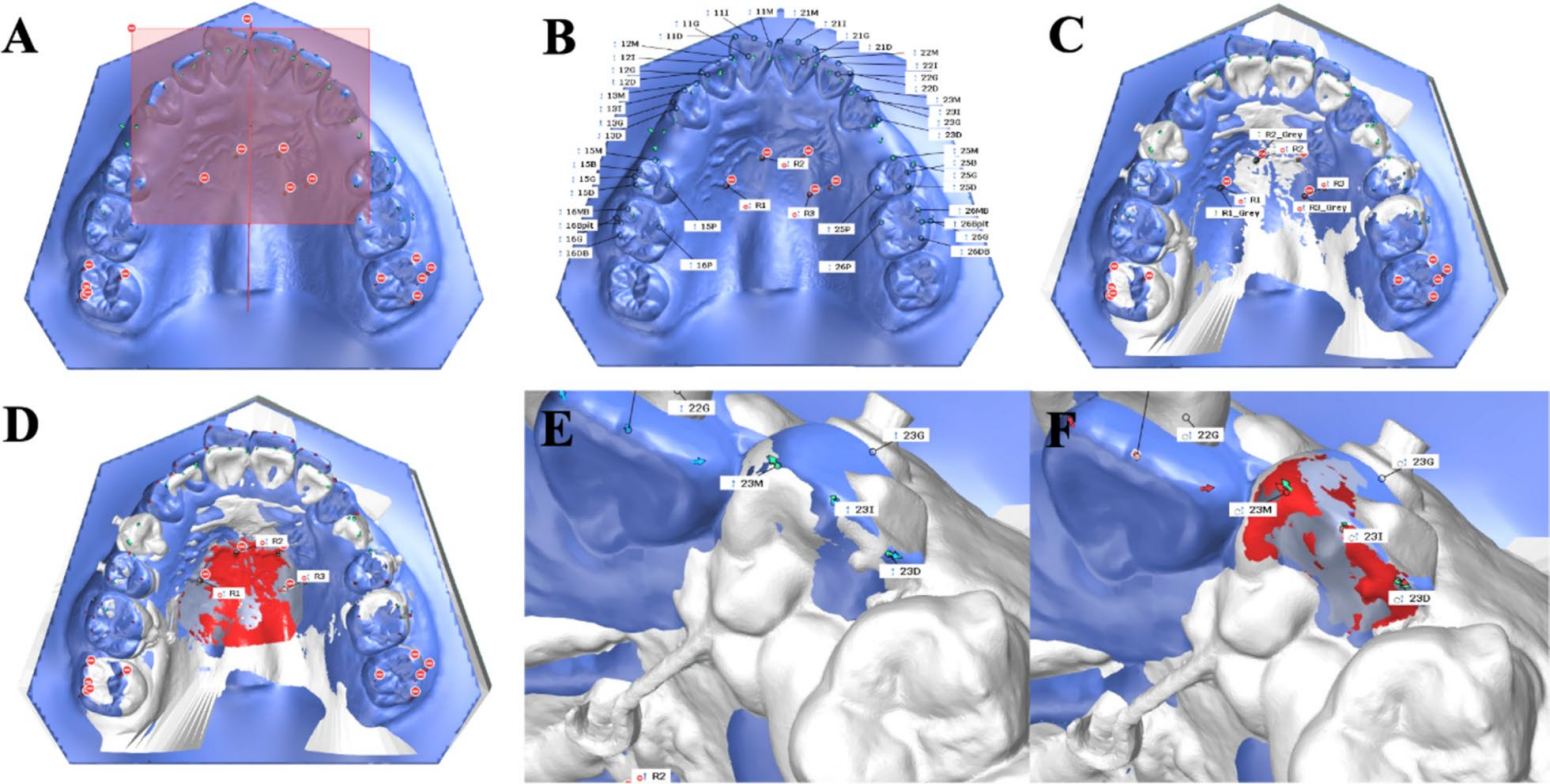
Superimposition method using GOM inspect suite The master files were imported to GOM inspect suite software; **A** Local coordinate system was constructed by fitting dental planes on the master model; **B** Each surface point was selected and defined; **C** Superimposition of the actual model with the master file(pretreatment), initially using the 3-points alignment method; **D** Followed by the local best-fit function at stable palatal area; **E** Superimposition of the canine, first by using the Geometric element method, then by local best-fit; **F** Superimposition of premolar and molar using local best-fit; The software would automatically transfer the landmark points from the master file and link to the actual file

The superimposition between the pretreatment and the completion of the canine final aligner (both virtual and actual) was modified from previous research [23, 27]. Details regarding the landmark points, palatal superimposition points, and the reference line on the model are presented in Table 4.

The details of superimposition steps are shown in Fig. 3. The virtual and actual models were superimposed using the palatal rugae and palatal vault as the reference area. For superimposing canines, we employed the Geometric Element method followed by the local best-fit method due to the significant distance change. For premolars and molars, only the local best-fit method was used.

#### Inspection

The GOM Inspect Suite software reported the linear (mm) and angular (°) movement difference values between the pretreatment stage and after completing the canine final aligner (virtual and actual).

The outcome data were collected from maxillary canines, second premolars, and first molars. The virtual power arm (VPa), virtual control (VC), actual power arm (APa), actual control (AC), and the difference of deviation (APa-VPa, AC-VC) were collected.

#### Linear measurements (Table 4 and Fig. 4A)

**Fig. 4:**
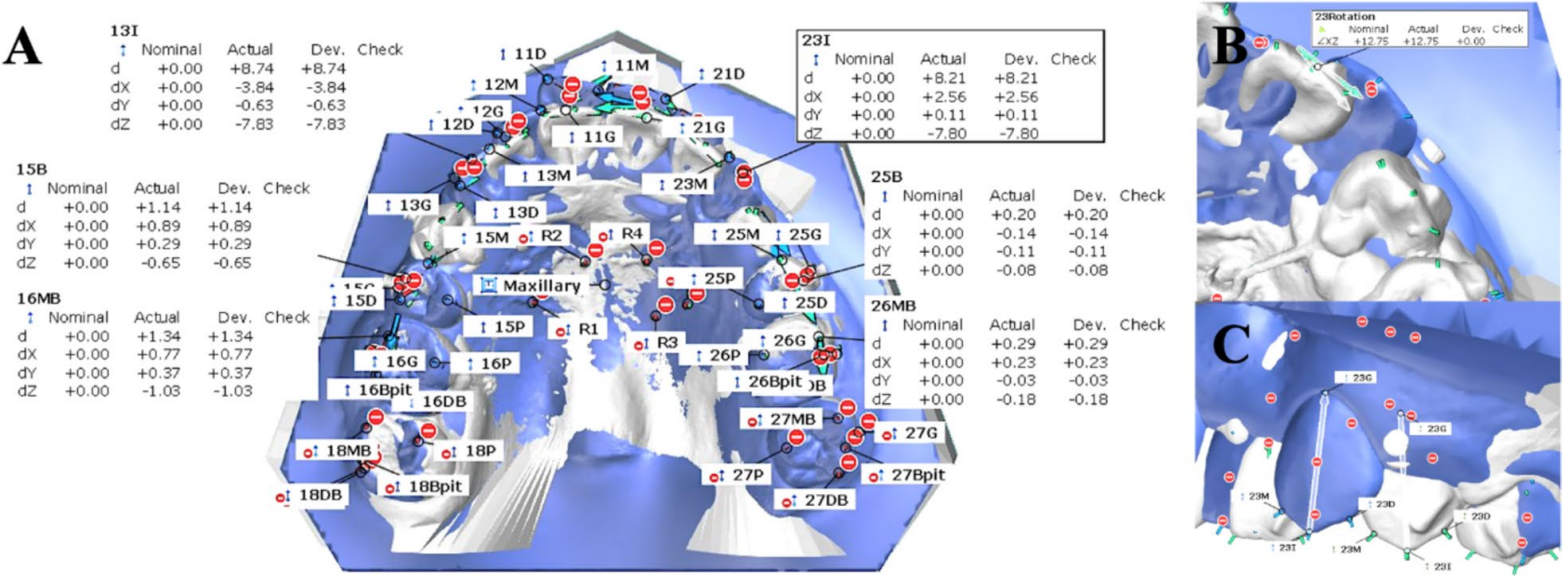
Measurement of surface point deviation (dX, dY, dZ) and tooth angulation between virtual and actual models **A** Linear measurement; **B** Rotation measurement between mesio-distal lines; **C** Tip and torque measurement between the tooth axis lines

The cusps of canines, buccal cusps of second premolars, mesio-buccal cusps of first molars, were used as a reference point to measure the tooth displacement. Data collected on both the power arm and control sides were mesialization-distalization, extrusion-intrusion, and buccal-lingual distance.

#### Angular measurements (Table 4 and Fig. 4B, C)

Angular measurements were measured from the mesio-distal and occlusal-cervical of the tooth axis, which included the tip, rotation, and torque changes.

All measurements were recorded in an Excel spreadsheet for statistical analysis.

### Sample size calculation

Using data from our pilot study and accounting for the randomized split-mouth design, a paired-sample framework was applied to calculate sample size. The calculation was performed using Minitab Statistical Software (Minitab, LLC, State College, PA, USA), based on canine distalization as the primary outcome, to achieve 80% power at a 5% two-sided significance level for detecting a mean paired difference in deviation of 0.31 mm. Assuming a standard deviation of 0.27 mm, an equivalence margin of ± 0.5 mm was defined according to a clinically acceptable range [27]. Accordingly, the minimum required sample size was 14 patient pairs. To ensure adequate power and allow for potential attrition, 21 participants were recruited.

## Interim analyses and stopping guidelines

None.

### Randomization

- Sequence generation

Sequence generation and randomization with a split-mouth design and a 1:1 allocation ratio were conducted using an online random number generator to assign the side of the maxillary canine and molar teeth (left or right). A sequence randomization list was created prior to the intervention.

b. Implementation

An independent resident, not involved in clinical treatment or outcome assessment, implemented the assignment of the palatal power arm and control according to the concealed randomization list.

### Blinding

Blinding of participants and operators was not feasible due to the prominent power arms; however, performance and detection bias were mitigated through standardized treatment protocols, independent outcome assessment, and landmark calibration.

### Statistical analysis

Outcomes for statistical analysis were mean differences between predicted (virtual) and achieved (actual) tooth movements for the PA and Control, expressed as (APa-VPa) – (AC-VC). The primary outcome was canine distalization; secondary outcomes included mesio-distal, bucco-lingual, extrusion-intrusion, tipping, rotation, and torque of canines, second premolars, and first molars.

### Equivalence testing [29]

All statistical analyses were performed using Minitab Online (Minitab, LLC, State College, PA, USA). Equivalence between the PA and Control was evaluated using the Two One-Sided Tests (TOST) procedure applied to the paired mean differences and standard deviations, with a p-value of 0.05 and 17 degrees of freedom. A priori equivalence margins were prespecified based on clinical relevance and measurement resolution (± 0.5 mm for linear outcomes and ± 1.5° for angular outcomes) [27].

The TOST *p*-value was defined as the maximum of the lower and upper one-sided p-values, as equivalence can only be claimed when both one-sided tests are statistically significant at the specified α level.

In addition, for each outcome, a 90% confidence interval (CI) of the mean difference was computed. Equivalence was concluded when the entire 90% CI lay within the prespecified equivalence bounds, which is mathematically equivalent to rejection of both one-sided null hypotheses of non-equivalence.

### Multiple-testing correction using the Holm–Bonferroni step-down procedure

Because multiple outcomes were evaluated within each tooth type and two tooth movement domains (3 linear or 3 angular movements), the family-wise error rate (FWER) was controlled using the Holm–Bonferroni step-down procedure with the number of tests within each family defined as *m* = 3 per tooth type and domain. Holm-adjusted thresholds are shown in Fig. 6.

TOST p-values were ranked from smallest to largest, and sequentially compared with its corresponding Holm-adjusted thresholds.

### Difference statistic test

Using the SPSS software, paired t-tests (for parametric) and Wilcoxon signed-rank tests (for non-parametric) were used to test for any significant differences in tooth movement changes and differences between the two sides.

### Power analysis

A post hoc power analysis for the equivalence test (canine distalization) was conducted using the two one-sided tests (TOST) procedure with paired data in Minitab software (Minitab LLC, State College, PA, USA). The analysis was based on the mean paired difference and standard deviation of the primary outcome, with predefined equivalence margins (± 0.5 mm) and a significance level of 0.05. Power was estimated under the requirement that both one-sided tests must be statistically significant and that the corresponding confidence interval lies entirely within the equivalence margins.

### Error analysis

Landmark identification and the assessment of discrepancies between the virtual and actual digital models were carried out for three linear displacements and three angular measurements. This process was repeated across all six pairs of digital models. Measurement precision was then evaluated using Dahlberg’s error formula.

## Results

### Participants flow

**Fig. 5:**
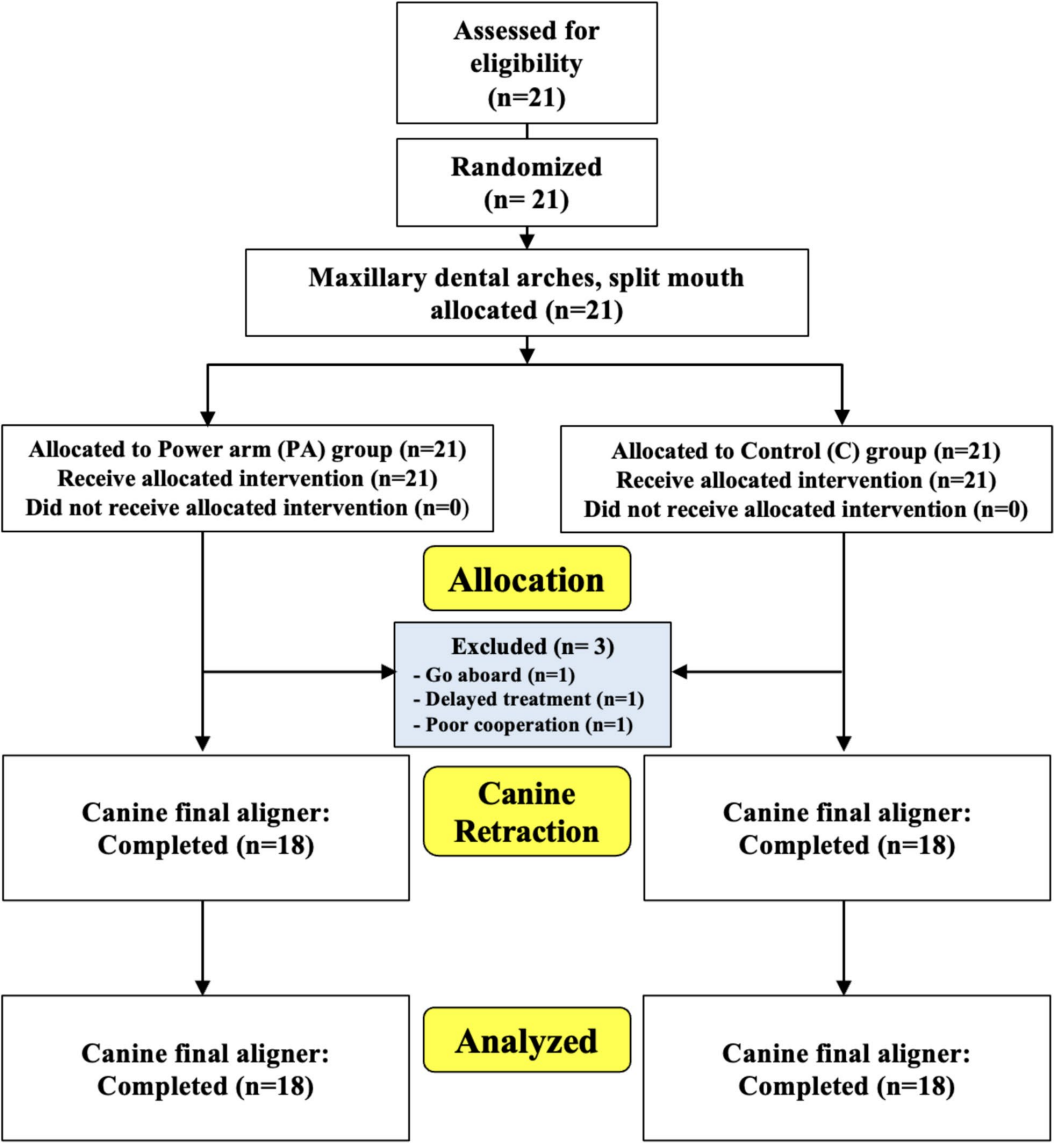
CONSORT flow diagram

The CONSORT flow diagram is presented in Fig. 5. Continuing the same clinical research done previously by Eurutairat et al. [27]. Initially, 21 patients were recruited and randomized for treatment. However, data from three patients were not collected for deviation analysis owing to the reasons shown in Fig. 5. Adult orthodontic patients were enrolled in this clinical study from 2020 to 2023.

### Baseline data

Demographic and baseline data for the patients are presented in Table 5. Final data were collected from 18 patients (1 male and 17 females), with a mean age of 23.46 ± 4.62 years old. Six patients were Angle’s class I, and 12 were class II. The mean maxillary crowding was -3.42 ± 1.50 mm, and the overjet was 6.10 ± 1.69 mm [27]. Descriptive symmetry analysis of left and right maxillary canines in anteroposterior position, rotation, and mesiodistal angulation is demonstrated in Appendix Table 1.

**Table 5:** Patient characteristics

Parameter	Mean (SD) / Frequency
Gender	F:M – 17:1
Age	23.46 ± 4.62 years old
**Angle’s Classification**	
Class I	6 cases
Class II division 1	12 cases
**Skeletal classification**	
Skeletal type I	3 cases
Skeletal type II	15 cases
Overjet Overbite	6.10 ± 1.69 mm 3.70 ± 1.86 mm
Crowding maxillary arch	−3.42 ± 1.50 mm
**Extraction pattern**	
U4s	4 cases
U4s—L4 or 5	12 cases
U4s – 1 lower incisor	2 cases
Lower fixed appliance	6 cases
Total number of aligners	24–50 aligners (12–25 Models)
Treatment time	15.75 months
**Off-track amount**	
None	3 cases
Mild ($\le$ 0.5 mm)	7 cases
Moderate (0.5-1 mm)	6 cases
Severe ($>$ 1 mm)	2 cases
**Off track management**	
None	14 cases
Back track	0 case
Elastic traction	3 cases
Rescan	1 case

### Numbers analyzed

The participant number analyzed (w/denominators) was 18/21 (85.7%) for each side (Fig. 5).

### Outcomes and estimation

Equivalence for a given outcome was declared statistically significant only when both criteria were satisfied:

(i) The TOST p-value was less than or equal to its corresponding Holm step-down adjusted α threshold, and
(ii) The 90% confidence interval lay entirely within the prespecified equivalence margins.

**Fig.6:**
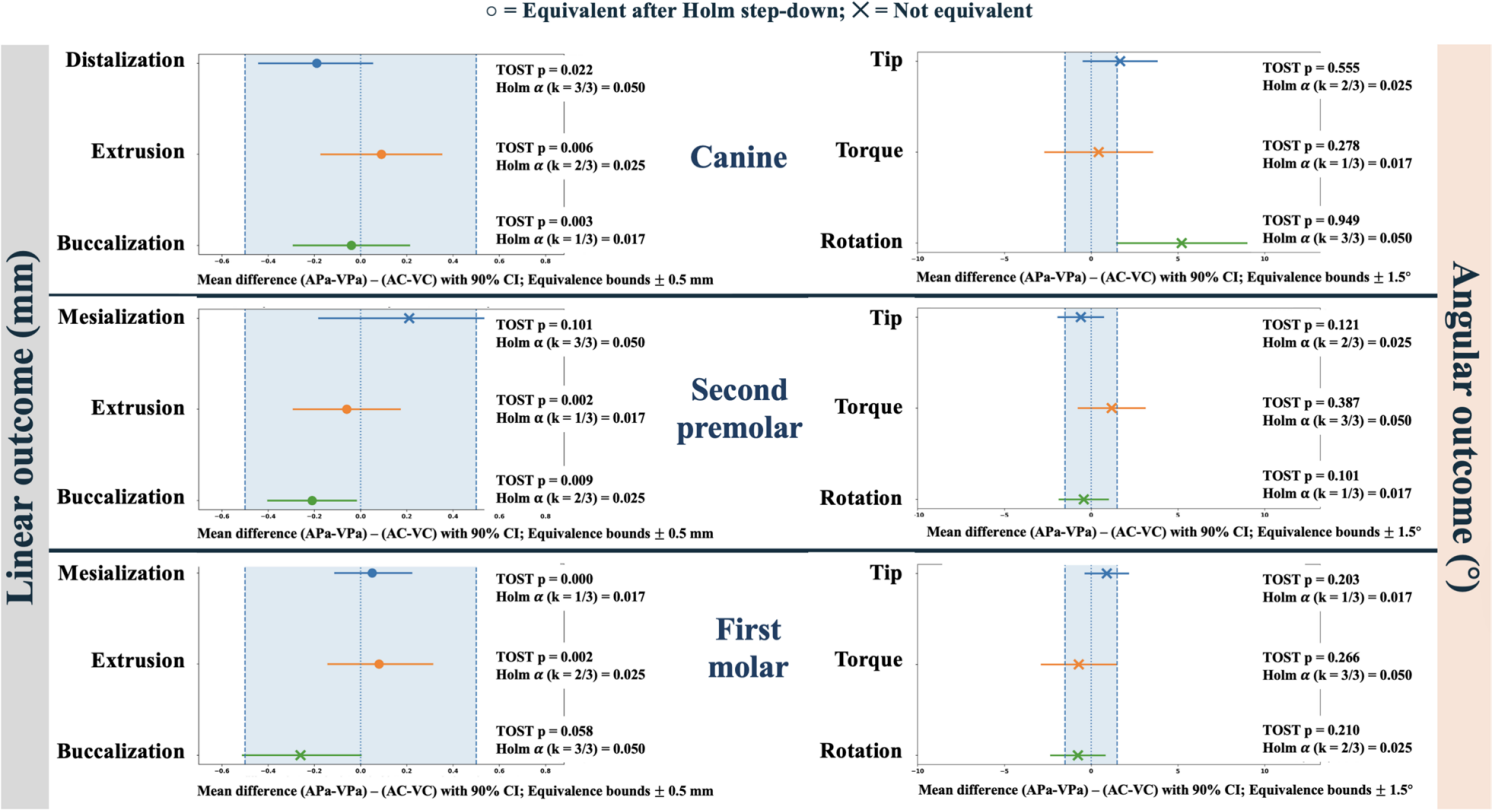
Equivalence (TOST) with Holm step-down threshold (m = 3) Abbreviation; Two One-Sided Tests (TOST), number of comparisons in the Holm family(m), virtual-power arm (VPa), virtual-control (VC), actual-power arm (APa) and actual-control (AC), confidence interval (CI), Holm adjusted alpha (Holm$\upalpha$), rank of the TOST *p*-value within the family of Holm step-down (k)

The primary and secondary outcomes are presented in Tables 6, 7 and 8 and Fig. 6, reporting the mean, standard deviation (SD), standard error (SE), and 90% CI for linear and angular displacement, paired differences.

**Table 6:** Maxillary canine movement; Comparison of actual-virtual differences between PA and C side [(APa-VPa) - (AC-VC)]. Results of Equivalence test (paired) with Holm-Bonferroni correction and Original difference test (Paired t-test or Wilcoxon signed-rank test) (N=18)

	Difference (A-V) Mean (SD) [95% CI]		Difference (PA-C) (APa-VPa)- (AC-VC)				Equivalence test with Holm-Bonferroni correction			Original difference tests (Paired t-test / Wilcoxon signed-rank test)	
	APa-VPa	AC-VC	Mean	SD	SE	90% CI	TOST *p*-value	Holm *p*-value	Equivalence	95% CI	*p*-value
Distalization(mm) = + Mesialization(mm) = −	−0.23 ± 0.44 [−0.45, −0.01]	−0.04 ± 0.64 [-0.35, 0.28]		0.59	0.14	−0.44, 0.05	0.022	0.050	Y	−0.49, 0.10	0.18
Extrusion(mm) = + Intrusion(mm) = −	−0.02 ± 0.44 [−0.24, 0.20]	−0.11 ± 0.63 [−0.43, 0.20]	0.09	0.62	0.15	−0.17, 0.35	0.006	0.025	Y	−0.22, 0.40	0.55
Buccal(mm) = + Lingual(mm) = −	−0.10 ± 0.36 [−0.28, 0.07]	−0.06 ± 0.33 [−0.23, 0.10]	− 0.04	0.14	0.14	−0.29, 0.21	0.003	0.017	Y	−0.34, 0.27	0.79
Tip (°) MCT = + DCT = −	−7.49 ± 5.89 [−10.41, −4.56]	−9.15 ± 6.72 [−12.49, − 5.81]	1.67	5.14	1.21	−0.44, 3.78	0.555	0.025	N	−0.87, 4.23	0.19
Torque (°) BCT = + LCT = −	0.76 ± 6.96 [−2.70, 4.23]	0.33 ± 3.80 [−1.56, 2.22]	0.43	7.51	1.77	−2.65, 3.52	0.278	0.017	N	−3.30, 4.17	0.81
Rotation (°) M-in = + D-in = −	−0.89 ± 7.50 [−4.61, 2.84]	−6.10 ± 6.15 [−9.16, -3.04]	5.22	9.11	2.15	1.48, 8.95	0.949	0.050	N	0.69, 9.75	0.03*

Abbreviation: VPa = Power arm side of virtual model; VC = Control side of virtual model; APa = Power arm side of actual model; AC = Control side of actual model; APa-VPa = Difference between actual and virtual model on power arm; AC-VC = Difference between actual and virtual model on control; # = Non- parametric statistics; TOST = Two One-Sided Tests [Y = Significant (Equivalence) at α = 0.05; N = Non-significant (Non-equivalence)]; Significance (Difference test)[ * Paired *t* test (for parametric)/ δ Wilcoxon signed-rank tests (for non-parametric), significant at *P* < 0.05.]

Favorable movement =  + (Distalization, Extrusion, Buccal, Mesial Crown Tip (MCT), Buccal Crown Torque (BCT), Mesial-in rotation)

Unfavorable movement =−(Mesialization, Intrusion, Lingual, Distal Crown Tip (DCT), Lingual Crown Tip (LCT), Distal-in rotation)

**Table 7:** Maxillary second premolar movement; Comparison of actual-virtual differences between PA and C side [(APa-VPa)—(AC-VC)]. Results of Equivalence test (paired) with Holm-Bonferroni correction and Original difference test (Paired t-test or Wilcoxon signed-rank test) (N = 18)

	Difference (A-V) Mean (SD) [95% CI]		Difference (PA-C) (APa-VPa)- (AC-VC)				Equivalence test with Holm-Bonferroni correction			Original difference tests (Paired t-test / Wilcoxon signed-rank test)	
	APa-VPa	AC-VC	Mean	SD	SE	90% CI	TOST *p*-value	Holm *p*-value	Equivalence	95% CI	*p*-value
Distalization(mm) = + Mesialization(mm) = −	− 0.85 ± 0.58 [−1.14, −0.56]	−1.05 ± 0.75 [−1.42, −0.68]	0.21	0.94	0.22	−0.18, 0.59	0.101	0.050	N	−0.26, 0.67	0.37
Extrusion(mm) = + Intrusion(mm) = −	−0.52 ± 0.50 [−0.77, −0.27]	−0.46 ± 0.56 [−0.73, −0.18]	− 0.06	0.57	0.13	−0.29, 0.17	0.002	0.017	Y	−0.34, 0.22	0.66
Buccal(mm) = + Lingual(mm) = −	-0.58 ± 0.42 [−0.79, −0.37]	−0.37 ± 0.33 [−0.54, −0.20]	−0.21	0.47	0.11	−0.40, 0.02	0.009	0.025	Y	−0.44, 0.03	0.06#
Tip (°) DCT = + MCT = −	−6.62 ± 3.60 [−8.41, − 4.83]	−6.02 ± 3.52 [−7.77, −4.27]	− 0.60	3.15	0.74	−1.89, 0.69	0.121	0.025	N	−2.17, 0.97	0.43
Torque (°) BCT = + LCT = −	−0.81 ± 4.71 [−3.16, 1.53]	−1.20 ± 4.78 [−4.37, 0.38]	1.18	4.65	1.10	−0.73, 3.09	0.387	0.050	N	−1.13, 3.49	0.30
Rotation (°) D-in = + M-in = −	−1.04 ± 1.86 [−1.97, −0.11]	−0.61 ± 3.02 [−2.12, 0.89]	− 0.43	3.42	0.81	−1.83, 0.97	0.101	0.017	N	−2.13, 1.27	0.60

Abbreviation: VPa = Power arm side of virtual model; VC = Control side of virtual model; APa = Power arm side of actual model; AC = Control side of actual model; APa-VPa = Difference between actual and virtual model on power arm; AC-VC = Difference between actual and virtual model on control; # = Non- parametric statistics; TOST = Two One-Sided Tests [Y = Significant (Equivalence) at α = 0.05; N = Non-significant (Non-equivalence)]; Significance (Difference test)[ * Paired *t* test (for parametric)/ δ Wilcoxon signed-rank tests (for non-parametric), significant at *P* < 0.05.]

Favorable movement = + (Distalization, Extrusion, Buccal, Distal Crown Tip (DCT), Buccal Crown Torque (BCT), Distal-in rotation)

Unfavorable movement = — (Mesialization, Intrusion, Lingual, Mesial Crown Tip (MCT), Lingual Crown Tip (LCT), Mesial-in rotation)

**Table 8:** Maxillary first molar movement; Comparison of actual-virtual differences between PA and C side [(APa-VPa)—(AC-VC)]. Results of Equivalence test (paired) with Holm-Bonferroni correction and Original difference test (Paired t-test or Wilcoxon signed-rank test) (N = 18)

	Difference (A-V) Mean (SD) [95% CI]		Difference (PA-C) (APa-VPa)- (AC-VC)				Equivalence test with Holm-Bonferroni correction			Original difference tests (Paired t-test / Wilcoxon signed-rank test)	
	APa-VPa	AC-VC	Mean	SD	SE	90% CI	TOST *p*-value	Holm *p*-value	Equivalence	95% CI	*p*-value
Distalization(mm) = + Mesialization(mm) = −	-0.86 ± 0.42 [− 1.07, − 0.65]	−0.91 ± 0.60 [-1.21, -.62]	0.05	0.40	0.09	−0.11, 0.22	0.000	0.017	Y	−0.15, 0.25	0.60
Extrusion(mm) = + Intrusion(mm) = −	−0.48 ± 0.41 [−0.68, −0.27]	−0.56 ± 0.39 [−0.75, -0.36]	0.08	0.55	0.13	−0.14, 0.31	0.002	0.025	Y	−0.19, 0.36	0.54
Buccal(mm) = + Lingual(mm) = −	−0.52 ± 0.26 [−0.65, −0.39]	−0.26 ± 0.57 [−0.54, 0.02]	−0.26	0.61	0.14	−0.51, 0.00	0.058	0.050	N	−0.56, 0.04	0.06#
Tip (°) DCT = + MCT = −	− 4.15 ± 3.50 [−5.89 −2.41]	−5.05 ± 2.63 [−6.36, −3.74]	0.90	2.99	0.71	−0.33, 2.14	0.203	0.017	N	−0.59, 2.39	0.22
Torque (°) BCT = + LCT = −	−1.37 ± 2.90 [−2.81, 0.07]	−0.66 ± 3.14 [−2.22, 0.91]	−0.72	5.21	1.23	−2.85, 1.42	0.266	0.050	N	−3.31, 1.87	0.57
Rotation (°) D-in = + M-in = −	−0.75 ± 2.70 [−2.10, 0.59]	0.01 ± 2.30 [−1.13, 1.16]	−0.77	3.76	0.89	−2.31, 0.78	0.210	0.025	N	−2.64, 1.11	0.78#

Abbreviation: VPa = Power arm side of virtual model; VC = Control side of virtual model; APa = Power arm side of actual model; AC = Control side of actual model; APa-VPa = Difference between actual and virtual model on power arm; AC-VC = Difference between actual and virtual model on control; # = Non- parametric statistics; TOST = Two One-Sided Tests [Y = Significant (Equivalence) at α = 0.05; N = Non-significant (Non-equivalence)]; Significance (Difference test) [* Paired *t* test (for parametric)/ δ Wilcoxon signed-rank tests (for non-parametric), significant at *P* < 0.05.]

Favorable movement =  + (Distalization, Extrusion, Buccal, Distal Crown Tip (DCT), Buccal Crown Torque (BCT), Distal-in rotation)

Unfavorable movement =—(Mesialization, Intrusion, Lingual, Mesial Crown Tip (MCT), Lingual Crown Tip (LCT), Mesial-in rotation)

Figure 6 displays the results of the equivalence test (TOST) with the Holm–Bonferroni step-down procedure (m = 3) using the mean differences between PA and control, (APa-VPa)-(AC-VC), for three linear (Mesio-distal translation, Extrusion, Buccalization) and angular outcomes (tip, torque, rotation), with 90% confidence intervals (CIs) and equivalence margins of ± 0.5 mm for linear and ± 1.5° for angular outcomes.

### Canine

- Linear outcomes

*Distalization*:

The 90% CI lies entirely within the equivalence bounds, and the TOST *p*-value (0.022) is below the Holm threshold for its rank (α₃ = 0.050). Therefore, equivalence in distalization is demonstrated.

*Extrusion*:

The 90% CI is fully contained within ± 0.5 mm, and the TOST p-value (0.006) is below the Holm threshold for its rank (α₂ = 0.025). Hence, equivalence in extrusion is demonstrated.

*Buccalization*:

The 90% CI remains within the equivalence region, and the TOST p-value (0.003) is lower than the most stringent Holm threshold (α₁ = 0.017). Thus, equivalence in buccalization is demonstrated.

b. Angular outcomes

*Tip*:

The 90% CI and mean difference between PA and C (1.67 $^\circ$) crosses the upper equivalence bound (+ 1.5°), and the TOST p-value (0.555) exceeds the Holm step-down threshold for its rank (α₂ = 0.025). Therefore, equivalence in tipping is not demonstrated.

*Torque*:

Although the 90% confidence interval extends beyond the equivalence bounds and the TOST *p*-value (0.278) exceeds the Holm-adjusted threshold for its rank (α₃ = 0.017), the mean difference between PA and C (0.43$^\circ$) lies within the equivalence margin; therefore, statistical equivalence in torque cannot be formally concluded (inconclusive).

*Rotation*:

The 90% CI lies almost completely outside the equivalence bounds, with a substantial positive mean difference between PA and C (5.22 $^\circ$), and the TOST *p*-value (0.949) exceeds the Holm threshold for its rank (α₃ = 0.050). Hence, equivalence in rotation is not demonstrated.

### Second premolar

- Linear outcomes

*Mesialization*:

Although the TOST *p*-value (0.101) exceeds the Holm-adjusted threshold for its rank (α₂ = 0.050), the 90% confidence interval lies mostly within the equivalence bounds; therefore, statistical equivalence cannot be formally concluded (inconclusive).

*Extrusion*:

The 90% CI is fully contained within ± 0.5 mm, and the TOST p-value (0.002) is below the most stringent Holm threshold (α₁ = 0.017). Equivalence in extrusion is demonstrated.

*Buccalization*:

The 90% CI lies within the equivalence region, and the TOST p-value (0.009) is below the Holm threshold for its rank (α₂ = 0.025). Thus, equivalence in buccalization is demonstrated.

b. Angular outcomes

*Tip*:

Although the TOST *p*-value (0.121) exceeds the Holm-adjusted threshold for its rank (α₂ = 0.025), the 90% confidence interval lies mostly within the equivalence bounds; therefore, statistical equivalence cannot be formally concluded (inconclusive).

*Torque*:

Although the 90% confidence interval extends beyond the equivalence bounds and the TOST p-value (0.387) exceeds the Holm-adjusted threshold for its rank (α₃ = 0.050), the mean difference between PA and C (1.18 $^\circ$) lies within the equivalence margin; therefore, statistical equivalence in torque cannot be formally concluded (inconclusive).

*Rotation*:

Although the TOST *p*-value (0.101) exceeds the Holm-adjusted threshold for its rank (α₁ = 0.017), the 90% confidence interval lies mostly within the equivalence bounds; therefore, statistical equivalence cannot be formally concluded (inconclusive).

### First molar

- Linear outcomes

*Mesialization*:

The 90% CI lies entirely within ± 0.5 mm, and the TOST p-value (< 0.001) is below the Holm threshold (α₁ = 0.017). Equivalence in mesialization is demonstrated.

*Extrusion*:

The 90% CI is fully contained within the equivalence bounds, and the TOST *p*-value (0.002) is below the Holm threshold for its rank (α₂ = 0.025). Equivalence in extrusion is demonstrated.

*Buccalization*:

Although the TOST *p*-value (0.058) exceeds the Holm-adjusted threshold for its rank (α₃ = 0.050), the 90% confidence interval lies mostly within the equivalence bounds; therefore, statistical equivalence in buccalization cannot be formally concluded (inconclusive).

b. Angular outcomes

*Tip*:

Although the TOST *p*-value (0.203) exceeds the Holm-adjusted threshold for its rank (α₃ = 0.017), the 90% CI lies mostly within the equivalence bounds, and the mean difference between PA and C (0.9 $^\circ$) lies within the equivalence margin; therefore, statistical equivalence in tipping cannot be formally concluded (inconclusive).

*Torque*:

Although the TOST *p*-value (0.266) exceeds the Holm-adjusted threshold for its rank (α₃ = 0.050), the 90% confidence interval lies mostly within the equivalence bounds, and the mean difference between PA and C (–0.72 $^\circ$) lies within the equivalence margin; therefore, statistical equivalence in torque cannot be formally concluded (inconclusive).

*Rotation*:

Although the TOST *p*-value (0.210) exceeds the Holm-adjusted threshold for its rank (α₃ = 0.025), the 90% confidence interval lies mostly within the equivalence bounds and the mean difference between PA and C (−0.77 $^\circ$) lies within the equivalence margin; therefore, statistical equivalence in rotation cannot be formally concluded (inconclusive).

### Difference test

Statistically significant differences in deviation were observed between the PA (APa-VPa –0.89 ± 7.50°) and the control side (AC-VC −6.10 ± 6.15°) in canine rotation, with a mean difference of 5.22 ± 9.11°.

### Power analysis

A post hoc power analysis for the equivalence test was performed using the two one-sided tests (TOST) procedure with paired data in Minitab software (Minitab LLC, State College, PA, USA). The analysis was based on the observed mean paired difference (0.19 mm) and standard deviation (0.59 mm), with predefined equivalence margins of ± 0.5 mm and a significance level of 0.05. The resulting statistical power was 68.8%.

### Error measurements

Dahlberg’s error analysis showed that all linear and angular measurements had discrepancies of less than 0.5 mm or 0.5° across all evaluated variables (Table 9).

**Table 9:** Error analysis performed using Dahlberg’s formula (n = 6)

Maxillary tooth	Dahlberg					
	Displacement			Angulation		
	A-P (mm)	Vertical (mm)	Transverse (mm)	Tip (°)	Torque (°)	Rotation (°)
Canine	0.15	0.18	0.09	0.20	0.16	0.11
Second premolar	0.09	0.09	0.08	0.19	0.19	0.12
First Molar	0.08	0.07	0.05	0.20	0.13	0.11

### Harms

At this stage, no harm was observed in any participant during the trial.

## Discussion

**Fig. 7:**
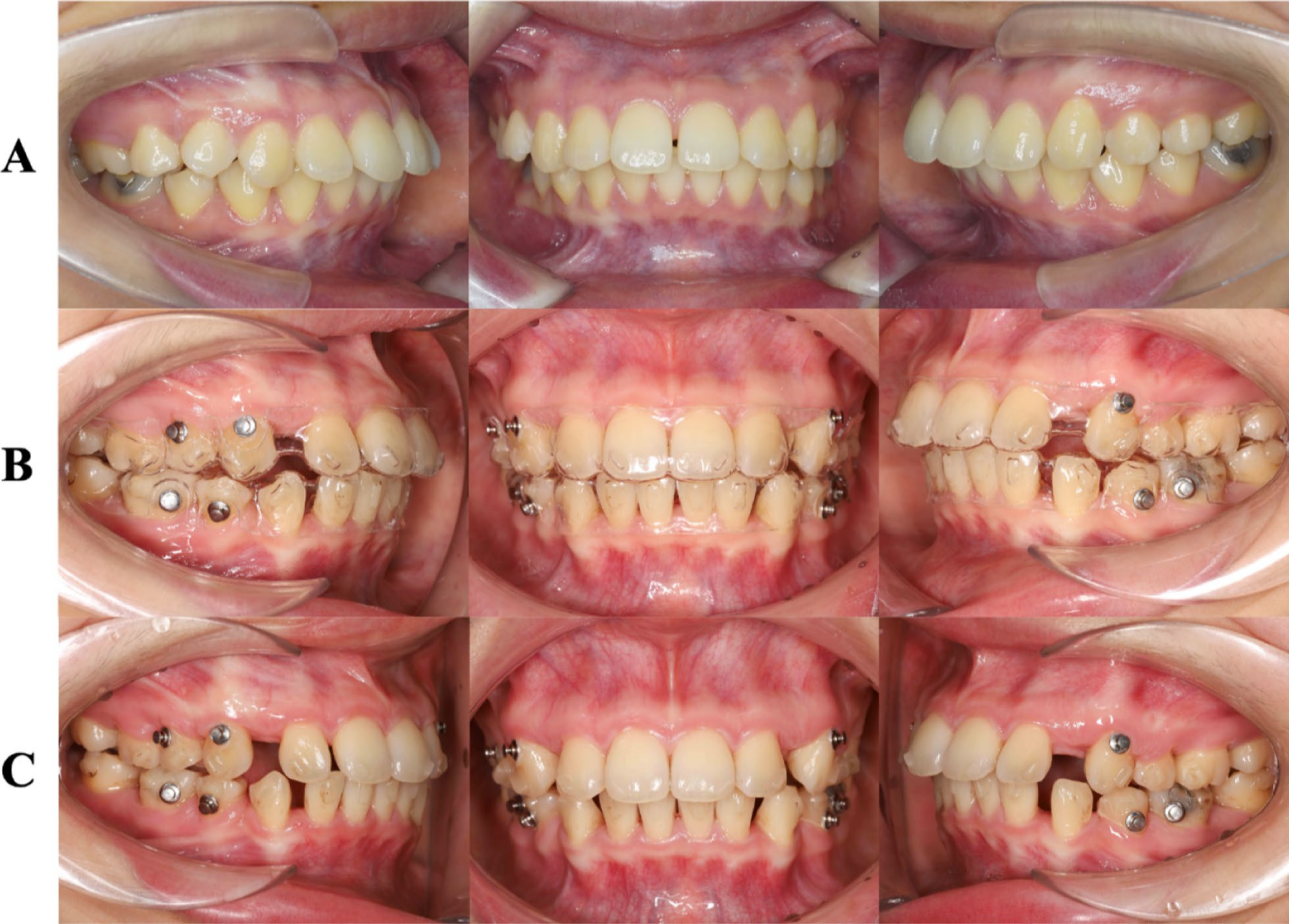
Intraoral photograph **A** Pretreatment stage; **B** In-house clear aligner at canine final retraction; **C** At canine final retraction without aligners

The present study evaluated the equivalence of tooth movements between PA and control approaches using a TOST framework with Holm step-down correction, interpreted through 90% CIs and predefined equivalence margins. The principal finding was that linear tooth movements were more likely to demonstrate equivalence than angular movements after adjustment for multiple comparisons. A representative case is illustrated in Fig. 7.

For the canine, all three linear movements (distalization, extrusion, and buccalization) met the equivalence criteria, whereas tip and rotation did not. However, equivalence in torque is inconclusive. A similar pattern was observed for the second premolar, in which extrusion and buccalization showed equivalence among linear outcomes. However, the equivalence of all angular movements is inconclusive. For the first molar, mesialization and extrusion demonstrated equivalence. Across all tooth types, angular outcomes showed wider CIs and more frequent violations of the equivalence bounds.

The 90% confidence intervals for canine tipping (− 0.44 to 3.78) and rotation (1.48 to 8.95) suggested non-equivalence. Interestingly, comparison of the mean differences between actual and predicted movements on the PA and control sides—canine tipping (APa–VPa: − 7.49 vs AC–VC: − 9.15) and canine rotation (APa–VPa: − 0.89 vs AC–VC: − 6.10)—showed greater discrepancies between the actual tooth movement from the virtual setup on the control side. Consequently, the paired mean differences [(APa–VPa) – (AC–VC)] for canine tipping (1.67°) and rotation (5.22°) may imply that canines on the PA side exhibited less tipping and rotation. We hypothesized that the PA might help create an anti-tipping and anti-rotational moment on the palatal side, thereby enhancing tipping and rotational control on the PA side.

A major strength of this study is its randomized controlled split-mouth design, which controlled pretreatment characteristics and ensured robust PICO-based comparisons. The multi-stage protocol allowed close monitoring and timely midcourse corrections. Deviation analysis was performed using GOM Inspect, enabling precise assessment of IHCA predictability through best-fit and three-point alignment with automatic virtual–actual tooth matching.

Direct comparison with prior clear-aligner studies is limited because earlier work relied on paired t-tests, correlations, or percentage accuracy, which assess differences or associations rather than clinical agreement. As noted by Pandis and Burzykowski [29], these metrics do not establish accuracy or clinically meaningful agreement. In contrast, our equivalence-testing framework evaluates whether deviations fall within prespecified clinical margins, providing a more clinically interpretable assessment of aligner accuracy.

The clinical outcomes of in-house clear aligner (IHCA) therapy, particularly regarding maxillary canine control, remain incompletely characterized. To date, only two studies using commercial Invisalign systems have reported tooth movement accuracy following canine retraction [16, 21], while the limited available IHCA evidence has been restricted to the initial and intermediate phases of retraction [23, 27]. Besides, no previous study has directly compared the accuracy of power-arm–assisted canine movement with that of clear aligners.

It should be noted that the equivalence margins of ± 0.5 mm and ± 1.5° were adopted from prior aligner accuracy studies, as they represent differences that are generally considered clinically negligible. Linear discrepancies < 0.5 mm are typically not perceptible and rarely influence occlusion or treatment decisions, while angular differences within ± 1.5° fall within the range of minor variation (≈1–2°) that does not meaningfully affect tipping, rotation, or treatment outcomes [2, 3, 14, 30]. Therefore, deviations within 0.5 mm and 1.5° are unlikely to be clinically detectable or to impact occlusal results or stability, supporting their use as clinically relevant equivalence margins.

The present study evaluated outcomes at the completion of canine retraction, whereas previous studies [16, 18, 20] assessed full anterior retraction; however, similar patterns of tooth movement were observed. Consistent with prior Invisalign studies, canines exhibited less distalization than planned but greater tipping, rotation, and extrusion, while posterior teeth showed greater mesial displacement, tipping, and intrusion than predicted. These findings are in agreement with previous reports in premolar extraction cases treated with Invisalign by Dai et al. [14, 15, 20], Ren et al. [16], and Feng et al. [13], suggesting that these biomechanical tendencies may be common across clear aligner systems.

The results of our study add information to previously reported findings by Vongtiang et al. [23], Eurutairat et al. [27], Gaffuri et al. [17], and Womack [21], indicating that a palatal power arm may help control maxillary canine tipping during the final phase of canine retraction.

Prior Invisalign studies [13–16, 20] used a one-stage digital setup, which in extraction cases is prone to off-track errors during space closure. In contrast, the multi-stage protocol with interim rescanning and replanning allows biomechanical recalibration as the edentulous span decreases, improving tracking accuracy and reducing cumulative errors and refinements.

The post hoc power of 68% should be interpreted within the equivalence testing framework. The TOST procedure is inherently stringent, requiring both confidence interval bounds to fall within predefined margins, and the use of narrow margins (0.5 mm/1.5 degrees)further reduces the likelihood of declaring equivalence. Combined with variability in angular outcomes, this likely contributed to reduced statistical power. Consequently, the study may be underpowered to formally demonstrate equivalence despite small, clinically negligible differences, as acknowledged in the revised manuscript.

Additionally, the study population was restricted to patients with mild crowding and mild canine asymmetry requiring premolar extraction, enhancing internal validity but limiting recruitment and sample size. Three patient dropouts (18/21 completed) further reduced power. Despite these limitations, the study provides clinically meaningful insights within a rigorous and conservative equivalence framework.

## Limitations

Several limitations should be acknowledged. Treatment accuracy is influenced by patient-, laboratory-, and measurement-related factors. Differences in aligner material and thickness (PET-G 0.5–0.75 mm vs multilayer polyurethane 0.75 mm) may alter force delivery and biomechanical responses [6, 31–33]. The bite-block effect of aligners and the use of hybrid mechanics likely contributed to differential vertical molar changes between full IHCA and hybrid protocols [34–36]. Measurement error arising from scanning, software processing, 3D printing may also have affected precision [26].

Another limitation is the use of palatal rugae for digital model superimposition, which can remodel during anterior retraction [37–39]. Without skeletal anchorage (e.g., TADs), posterior teeth may not remain stationary, potentially affecting the accuracy of the best-fit superimposition.

Finally, patient compliance remains a critical determinant of the achieved clinical outcomes [27].

Although the prespecified equivalence margins were clinically justified, they are inherently context dependent. While adequately powered for the primary outcome, the study may have been underpowered to demonstrate equivalence for angular movements with greater variability. The analysis focused on mean paired differences and did not account for potential effect modifiers (e.g., tooth morphology, attachment design, biological responsiveness). The Holm step-down procedure, although appropriate for multiplicity control, increased the stringency of equivalence testing and may have led to conservative conclusions for outcomes near the equivalence bounds.

Future research suggestions include prospective clinical studies with larger sample sizes, using various aligner materials and wearing protocols, and incorporating 3D cone-beam computed tomography in the aligner setup and superimposition process for greater precision to achieve a more comprehensive understanding of IHCA.

## Generalizability

The generalizability of these findings is limited. Results may not be directly applicable to commercial aligner systems due to differences in laboratory processes and clinical protocols, and outcomes were assessed at the completion of canine retraction rather than full treatment. Additionally, the in-house setup protocol is system-specific; thus, caution is required when extrapolating to other systems with different materials, staging protocols, attachment designs, or auxiliary mechanics.

## Clinical recommendations

Clinically, palatal power arms may be considered to enhance angular control, particularly for managing canine rotation and tipping during extraction space closure.

## Conclusions

- The majority of linear tooth movements demonstrated equivalence between the PA and control sides.
- Angular movements, particularly canine rotation and distal crown tipping, were not equivalent.
- These findings suggest that while the PA and control sides are clinically comparable for translational movements, angular control of the canine may differ between the two sides.
- The use of a power arm may assist canine retraction by improving control of canine angulation; however, the evidence from this study should be interpreted with caution.
- The available evidence is insufficient to conclude the similarity in anchorage behavior between the two sides.

## Data Availability

The datasets used and/or analyzed in the current study are available from the corresponding author upon reasonable request.

## Appendix

See appendix Table 10

**Table 10:** Pretreatment symmetry analysis of right and left maxillary canines

	Mean difference	SD	95% CI
Anteroposterior	1.31 mm	0.64	0.99–1.63
Rotation	4.38°	4.99	1.90–6.86
Angulation	3.09°	4.29	0.96–5.22

^*^ Pretreatment digital model measurements assessing symmetry between right and left maxillary canines, including anteroposterior distance (cusp tips of 13 and 23), rotation relative to the midsagittal plane, and mesiodistal inclination relative to the occlusal plane, using GOM Inspect Suite and Photoshop

## Abbreviations

IHCA: In-house clear aligner
RCT: Randomized controlled clinical trial
IPR: Interproximal reduction
V: Virtual
A: Actual
P: Pretreatment
SI: Superimposition
PA: Palatal power arm
C: Control
VPa: Power arm side of virtual model
VC: Control side of virtual model
APa: Power arm side of actual model
AC: Control side of actual model
APa-VPa: Difference between actual and virtual model on power arm
AC-VC: Difference between actual and virtual model on control
MPa: Moment of palatal power arm
MA: Moment of aligner
TOST: Two one-sided tests
FWER: Family-wise error rate
